# How Delphi studies in the health sciences find consensus: a scoping review

**DOI:** 10.1186/s13643-024-02738-3

**Published:** 2025-01-14

**Authors:** Julia Schifano, Marlen Niederberger

**Affiliations:** Department of Research Methods in Health Promotion and Prevention, Institute for Health Sciences, University of Education Schwäbisch Gmünd, Oberbettringer Straße 200, Schwäbisch Gmünd, 73525 Germany

**Keywords:** Expert survey, Agreement, Health, Conducting, Reporting, Bias

## Abstract

**Background:**

Delphi studies are primarily used in the health sciences to find consensus. They inform clinical practice and influence structures, processes, and framework conditions of healthcare. The practical research—how Delphi studies are conducted—has seldom been discussed methodologically or documented systematically. The aim of this scoping review is to fill this research gap and to identify shortcomings in the methodological presentation in the literature. On the basis of the analysis, we derive recommendations for the quality-assured implementation of Delphi studies.

**Methods:**

Forming the basis of this scoping review are publications on consensus Delphi studies in the health sciences between January 1, 2018, and April 21, 2021, in the databases Scopus, MEDLINE via PubMed, CINAHL, and Epistemonikos. Included were publications in German and English containing the words “Delphi” in the title and “health” and “consensus” in the title or abstract. The practical research was analyzed for the qualitative content of the publications according to three deductive main categories, to which an influence on the result of Delphi studies can be imputed (expert panel, questionnaire design, process and feedback design).

**Results:**

A total of 287 consensus Delphi studies were included in the review, whereby 43% reported having carried out a modified Delphi. In most cases, heterogeneous expert groups from research, clinical practice, health economics, and health policy were surveyed. In about a quarter of the Delphi studies, affected parties, such as patients, were part of the expert panel. In the Delphi questionnaires it was most common for standardized Likert scales to be combined with open-ended questions. Which method was used to analyze the open-ended responses was not reported in 62% of the Delphi studies. Consensus is largely (81%) defined as percentage agreement.

**Conclusions:**

The results show considerable differences in how Delphi studies are carried out, making assessments and comparisons between them difficult. Sometimes an approach points to unintended effects, or biases in the individual judgments of the respondents and, thus, in the overall results of Delphi studies. For this reason, we extrapolate suggestions for how certain comparability and quality assurance can be achieved for Delphi studies.

**Supplementary Information:**

The online version contains supplementary material available at 10.1186/s13643-024-02738-3.

## Background

Delphi studies are used in the health sciences with the primary goal of finding consensus [1–3]. The aim is “to obtain the most reliable consensus of opinion of a group of experts” [4]. The concept of consensus is often understood to be the majority of the participants agreeing on a standardized item [5]. In healthcare, consensus is most frequently measured using percentage agreement [6, 7]. Zarnowitz and Lambros [8] define consensus as “the degree of agreement among point predictions aimed at the same target by different individuals” [8]. To reach a consensus, experts participating in a Delphi study evaluate concrete epistemic issues over multiple rounds [3, 9]. A crucial difference from one-time surveys is that the expert panel is not randomly selected and there is no claim of statistical representativity in the results [10].

In a classic Delphi study, the experts’ judgments are typically collected anonymously using (online) questionnaires [11]. However, researchers will sometimes modify the classic Delphi so that the survey process fits the goals or the resources available for the project. For instance, this can involve having participants meet face-to-face or limiting the number of rounds from the start [12–14]. In a systematic review of Delphi studies that identify healthcare quality indicators (*n* = 80), Boulkedid et al. [13] found that more than half of the Delphi studies reported following a modified Delphi. Yet, these modifications are not always described or justified [15, 16].

Now, alongside what often appear to outsiders to be nontransparent modifications are Delphi variants whose approaches are clearly articulated and justified. Among them are the policy Delphi [17] and real-time Delphi [18]. Some of the reasons for these developments are to better record the general context behind standardized judgments and to enable anonymous debates of the arguments in real time [17, 18]. However, only in individual cases have these different variants been reflected on or evaluated in terms of their methodology. Moreover, how modifications affect the overall results of Delph studies is still to the largest extent unclear. Evaluations of the real-time and classic Delphi found no significant difference in the overall result between the Delphi variants [19, 20]. However, the analysis by Quirke et al. [20] suggests that respondents are less likely to adjust their judgment in a real-time Delphi. When adjusted, it is more in the direction of the group mean than in the classic Delphi [20]. In our view, despite differences in study design, the following characteristics constitute a Delphi study [3, 4, 9]:

1. Survey of several people with specialized knowledge (known as experts) (e.g., operational knowledge, experiential knowledge, functional knowledge, contextual knowledge);
2. Carrying out at least two survey rounds or the option to respond at least two times;
3. Feedback and the (interim) results are presented to the respondents with a possibility to respond.

Typically, Delphi studies also share a focus on complex topics and questions that require a certain level of expertise and experience to answer [9]. The theoretical assumption underlying the Delphi technique is that multiple experts must be asked in order to cover different perspectives [21, 22]. To gather valid and practicable results, those conducting the Delphi must understand the response behavior of the experts, especially when the aim of the Delphi is to reach a consensus [23, 24]. This is because it has direct practical relevance, for instance, when consensus is sought to make concrete recommendations for routine clinical practice (e.g., [25]) or for medical curricula (e.g., [26]). Furthermore, Delphi studies are also very widespread in the field of health [27]. For these reasons, we want to shed light from a methodological perspective on how consensus is found in Delphi studies in the health sciences. However, first, we explain how judgments are formed in Delphi studies and which factors influence them.

### Theoretical insights on judgment formation in consensus Delphi studies

In standardized surveys, response behavior is described as an ideal type of process consisting of four steps: (I) understanding the question, (II) retrieving information, (III) evaluating the question, and (IV) submitting the response. Making the cognitive effort in all steps is described by Krosnick [28] as *optimizing*. The intensity of the effort at each step of the response process model depends, among other things, on the motivation and ability of the respondent and the difficulty of the task [28]. Depending on what influences these factors exert, the response strategy of *satisficing* can come into play [28]. When satisficing, the respondent engages more superficially with the steps of the response process or skips over individual steps entirely to give an answer to the question asked [28, 29].

**Fig. 1:**
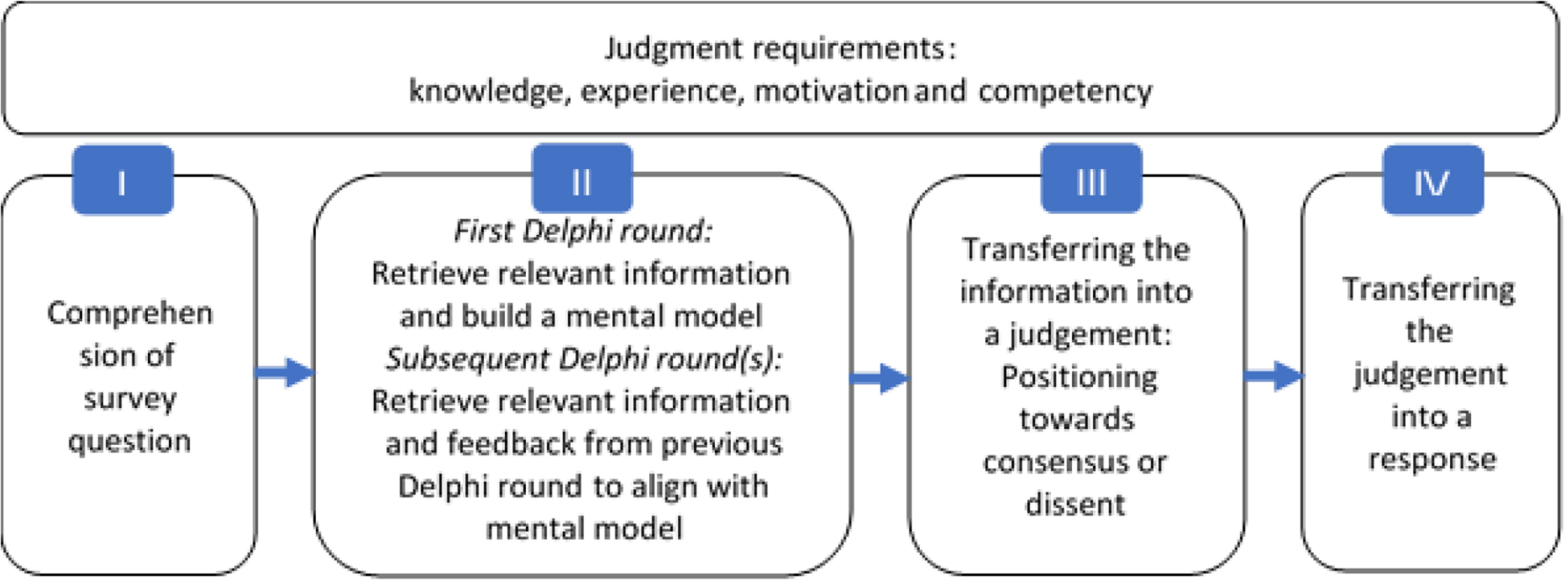
Theoretical process for reaching optimal judgments in consensus Delphi studies based on Tourangeau et al. 2000 [35]

From a cognitive psychology perspective, judgment formation in consensus Delphi studies can be augmented by the idea of mental models [22, 30]. With Delphi studies, it must be assumed that participants go through the steps of the response process in a state of cognitive uncertainty because, typically, uncertain and incomplete knowledge exists regarding the topics, which sometimes extend beyond the experts' main area of expertise [21, 22]. Specialized knowledge on the part of the respondents is required to comprehend, contextualize (step I, Fig. 1), and evaluate questions [3, 22]. When forming judgments (steps II and III, Fig. 1), experts are often required to place the question in a larger context and generate transformation knowledge beyond the scope of their specialty [22, 31], e.g., in regard to the consequences of the judgment for affected groups [32], future generations [33] or other specialized areas in an organization [34]. Furthermore, Delphi studies sometimes integrate additional information which the respondents should consider when forming their judgments, e.g., a summary of the current state of research [25]. Ideally, all of the information is taken into consideration by the respondents when forming judgments (see step II, Fig. 1).

From the second round onward, feedback plays another central role in the process of forming judgments [4, 11]. The difference from the first Delphi round is that the experts have already formed a mental model and they receive the feedback as additional information (step II, Fig. 1), which can consist of arguments put forth by other experts, statistical data regarding the group response, the expert’s response from the previous round, or a combination of these [7]. The feedback is meant to encourage the experts to include previously unconsidered aspects in their mental models in order to give a well-founded and carefully thought-out judgment according to the *optimizing* strategy [31, 36].

The individual process of coming to a judgment in a Delphi study is complex and can only function “optimally” under certain conditions (see “Judgment requirements”, Fig. 1), and, to be specific, if the respondents [30, 37]:

- Have extensive knowledge of the topic,
- Are familiar with the topic and have experience with it, meaning they regularly engage with the topic under investigation (usually for professional reasons, but also because they are personally affected),
- Have certain cognitive abilities and the motivation to specify, structure, and evaluate information.

In Delphi studies, judgments can also be influenced by other factors, such as the stated aim of finding consensus, thus making optimizing more difficult [38]. There is still little reflection on the theoretical level about how response behavior in Delphi studies takes shape in practice, even though this seems to be highly relevant. If there is little success in getting the experts to optimize their response process, the results will be less precise and less reliable [29]. *Satisficing* [28, 29] in Delphi studies could take these forms:

- Experts avoid a clear judgment on a specific position, e.g., in that they tend toward the middle of the scale or consciously take a decision that differs from the majority.
- Experts respond arbitrarily, e.g., in that they select the first answer on offer.
- Experts do not form a judgment, e.g., in that they leave out questions, choose an evasive category (e.g., “don’t know”), or discontinue the survey process.
- Experts form deliberate judgments but, consciously or unconsciously, do not consider all of the information equally, e.g., in that they only include the first response options or arguments in the qualitative feedback when forming their judgment.
- Experts respond such that the Delphi will be terminated, e.g., in that they more or less agree with the majority opinion as presented in the feedback in order to support a statistical consensus.

Contrary to the process outlined in the ideal model (Fig. 1), evidence shows that judgment formation is subject to suboptimal conditions that can make *optimizing* more difficult [29]. Hence, respondents’ individual personal characteristics, the situation, or the questionnaire's content and visual presentation have effects on response behavior and thus on the overall results. In the following, we present an overview of methods studies that shed light on these aspects and show the effects on individual judgments.

### Methodological findings on judgment formation in Delphi studies

Different methodological findings exist in regard to how respondents form their judgments in Delphi studies, namely:

- Systematic reviews based on publications of Delphi studies [1, 2, 6, 7, 14, 16, 39]
- Method experiments [24, 34, 38, 40–44]
- Evaluation studies [23, 45–48]
- Reports by Delphi practitioners [11, 49, 50]

**Fig. 2:**
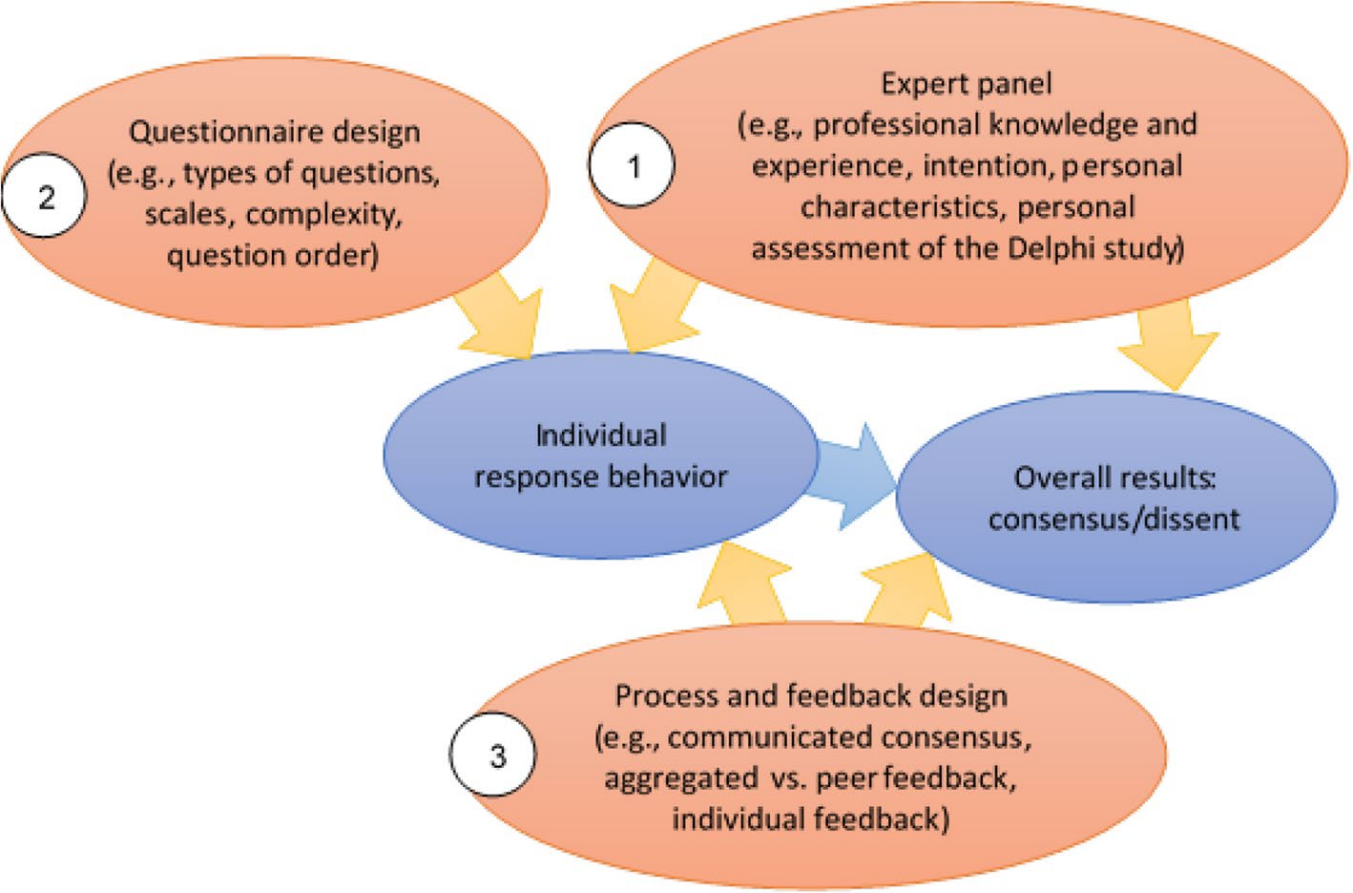
Factors influencing the results of consensus Delphi studies in the health sciences

According to these findings, three factors have a direct or indirect influence on the overall result of a Delphi study: the expert panel, the questionnaire design, and the process and feedback design (Fig. 2). How these three factors exert influence on individual response behavior and the overall result of a Delphi study is explained in the following.

1. The *expert panel* is a central feature of the Delphi technique. Empirical evidence demonstrates that five aspects affect the individual judgments of the respondents:
  The subjective perception of the baseline (e.g., estimation of the topic's relevance, majority opinions in the field) [41, 42, 48]
  The actual professional knowledge and experience (e.g., knowledge about current studies, position in an organization, lifeworld experiences) [23, 32, 51–53]
  The intention to participate in the Delphi study (e.g., personal and/or institutional interests and objectives) [23, 48]
  Personal characteristics (e.g., value systems) and sociodemographic profile (e.g., age) [23, 32, 43, 52]
   The assessment of the Delphi study (e.g., relevance or clarity of the study's aims and the Delphi technique) [46, 47]

Although it must be assumed that, along with expertise, these diversity variables have an effect, they are generally not considered in the selection of experts for Delphi studies [52]. Selection is typically done on the basis of professional expertise, e.g., through professional associations [16, 25]. Furthermore, the nature and size of the expert panel composition are relevant to the process of finding consensus. To ensure sufficient professional heterogeneity, it is recommended that experts be recruited through purposive sampling and not snowballing [10, 11]. How large a Delphi panel should be is not determined in terms of method, usually, the number is in the low double-digits [7]. Statistical models demonstrate that groups of this size can deliver stable final results, which depend, of course, on the topic and composition of the expert panel [54, 55]. When different expert groups, e.g., different disciplines, are included and the numbers between them are unequal, biases can emerge in favor of the judgments from the expert group with the most members. Beiderbeck et al. [56] therefore advise performing subgroup analyses if there are 15 to 20 members per expert group.

Generally speaking, heterogeneous panels reach consensus overall for fewer aspects of a question than homogeneous panels [34, 53]. Still, the heterogeneity of the expert panel regarding professional knowledge counts as quality enhancing for Delphi studies, despite the partially unclear influences on the overall study results [14, 16, 49, 50]. Influences arising from the individual personalities of the respondents carry less weight as a result [49].

2. With *questionnaire design*, we mean how questions in Delphi studies are (a) presented visually and in terms of content and sequence and (b) designed methodologically, e.g., what types of questions (open/closed) and scales. Bias in the filling out of a questionnaire, as is seen with every standardized survey, can also be avoided in Delphi studies by adhering to the recommendations for designing standardized questionnaires [57].

- Potential biases due to how questions and responses are formulated and presented can be mitigated in Delphi studies [49]. The background here appears to be that experts (including their mental models) analyze the questions more cognitively than citizens do with questions in survey polls [58].
  Nevertheless, a questionnaire's complexity plays an important role in Delphi studies [59]. An item's length should not exceed 25 words according to a recommendation from futures research [60]. Markmann et al. [24] found that longer and more abstract statements make the formation of individual judgments in Delphi studies more difficult and lead to more moderate judgments.
  Brookes et al. [61] investigated the effect of biases on judgments in Delphi studies arising from the order in which the questions are asked by presenting topic blocks to the participants in different sequences. By doing so, they were able to determine effects on the judgments of patients and healthcare professionals which were equally relevant to the overall result but could have different effects on it. Brookes et al. [61] and also Hallowell & Gambatese [62] therefore recommend randomizing the questions in Delphi studies, which has been reported by several studies (e.g., [63, 64]).

b) The relevance of open questions varies in regard to Delphi studies. Standardized items dominate mostly, and open comments are used only in individual cases or in an initial qualitative round [10, 65]. However, there are also Delphi studies that focus on the interchange of arguments from open comments [66]. The aim of free-text responses can be to supplement or specify details or to justify or appraise the judgments [65]. The problem, though, is that the handling and analysis of free-text responses is often not undertaken systematically [65]. In these cases, it is questionable if the increased cognitive effort required can be justified to the respondents [67].
  The study findings are unclear on scale range and the design of rating scales in Delphi studies. Different reviews show that rating scales are typically used to measure consensus in Delphi procedures and often have five or more graduations [6, 7, 16, 39]. Based on the results of their review of Delphi studies in histopathology, Taze et al. [16] recommend the use of a "nine-point Likert scale with a 'no opinion' option and a free-text comment box" [16]. Initial analyses indicate that scales with different lengths lead to a different end result [68, 69]. In a comparison of three scale lengths (3-point, 5-point, and 9-point rating scales), Lange et al. [68] determined that the 5-point rating scale with a cut-off value of 75% achieved the least consensus and the 9-point scale the most. It must be noted that, while the scales had the same defined cut-off value, different numbers of scale points were included in the definition of consensus [68]. De Meyer et al. [69] found higher consensus with a longer scale, whereby consensus was also defined here using one (3-point scale) or more (9-point scale) scale points. Both studies concluded that recommendations for direct action in clinical practice can be derived with a 3-point scale (e.g., “main goal,” “secondary goal” and “no goal”) and the result is simpler to interpret than with longer scales [68, 69].

3. Another factor influencing individual judgments and the overall result is *process and feedback design****. ***This influence is seen on three levels: a) the communicated consensus, b) the aggregated feedback and c) the individual feedback on a participant's response from the previous Delphi round.
  - Barrios et al. [38] differentially analyzed the influence of the level of agreement on the individual judgments and observed that if the consensus was over 75%, participants were more likely to converge with the opinion of the group than if the group's aggregated agreement was below this value [38]. Signs of a conscious judgment against the consensus were observed by Barrios et al. [38] when the value in the feedback lay below the consensus level of the percent agreement. They speculate that experts consciously manipulate results as a result of revealing the consensus [38]. Although this influence is theoretically probable, we are not aware of other publications on the effect of disclosing the level consensus or dissent on individual judgments, e.g., in the communication of the Delphi study's aims or as part of the feedback.
  - Feedback involving the statistical group response dominates in Delphi studies, while peer feedback is less frequently given [7]. The extent to which the form and type of feedback (qualitative or quantitative) influences judgment behavior is controversial [12, 41, 49]. Some Delphi practitioners support the use of qualitative instead of quantitative feedback because then the experts do not prematurely side with the majority opinion (bandwagon effect) [49, 50]. However, this assumes that the open responses are not presented in an unfiltered form, but rather systematically analyzed, which, as already described, is not always the case [49, 65]. In addition, the effects of differentiating the feedback by expert group have already been shown [40]. Brookes et al. [40] have demonstrated that, if the feedback contains information on different groups of participants, the level of agreement between the expert groups increases compared to peer feedback. MacLennan et al. [70] have also carried out a randomized Delphi study with different feedback strategies but were unable to confirm the effects observed by Brookes et al. [40]. Fish et al. [43] assert the hypothesis that in comparison to healthcare professionals, patients less often integrate the feedback of other expert groups and hence do not reflect as much on judgments made from other perspectives [43]. Turnbull et al. [45] show similar findings.
  - A randomized experimental study on urban sustainability by Meijering & Tobi [44] demonstrated that experts less often adjust their judgment when they see their response from the previous round; however, an effect on the final consensus could not be determined [44].

We are not aware of analyses of other factors affecting the process and feedback design, e.g., how the termination criterion or the numbers of rounds influence individual judgments and the overall result. Having said this, though, the termination criterion and the number of rounds are relevant in order to ascertain whether the consensus is stable and valid [1, 2].

### Aims of the scoping review

The methodical tests, experiments, and discussions presented here concerning Delphi studies in the health sciences ultimately identify three factors that can be alleged to exert an influence on the results of a Delphi and thus on the consensus: the expert panel, questionnaire design, and process and feedback design (Fig. 2). These three factors serve as the basis for the present scoping review. The aim is to highlight the range of methodological approaches in relation to these three factors and to identify the areas that have received little attention [71].

The following research question is answered in this scoping review:

How are the influential factors described here used in the practice of consensus Delphi studies in the health sciences?

In addition to these three proven factors, there are indications of other factors that influence individual judgments and the overall results of Delphi studies, e.g., the effect of the time between rounds [43], though such factors have not yet been fully examined explicitly for Delphi studies, e.g., the effect of sponsors or members of a supervisory group on the overall result. In general, a decrease can be seen in publications that examine Delphi studies in terms of methodology compared to Delphi primary studies [27]. Flostrand et al. [27] showed that the ratio of methodological studies to Delphi primary studies was 1:1 in 1975 and 1:19 in 2016. Other factors will not be considered in this scoping review due to a lack of evidence. In addition to the three proven factors, we identify general criteria for describing Delphi studies, including the Delphi variants and the definition of consensus. It must be noted that this review of research practice is based on publications of Delphi studies even though a lack of clarity and sometimes even errors have been repeatedly shown to exist in such publications [5, 7, 14]. Based on the analysis of research practice and taking into account theoretical and methodological findings on judgment building in Delphi studies, we draw conclusions for the quality-assured implementation of Delphi studies.

## Method

The reporting in this review is based on the PRISMA Extension for Scoping Reviews (PRISMA-ScR) [71]. This methodological approach has been discussed at different times with members of the German-speaking Delphi expert network (DEWISS), which is comprised of over 20 academics from various disciplines in the health and social sciences and epistemological approaches. DEWISS receives funding from the German Research Foundation (Deutsche Forschungsgemeinschaft, DFG), an interdisciplinary institution promoting science and research in Germany (project number 429572724, more information is available at https://delphi.ph-gmuend.de/). No study protocol was published.

### Search strategy and study selection

The search for publications was conducted on the basis of an already existing collection of 7,044 Delphi primary studies compiled by the Delphi expert network (available via https://www.zotero.org/groups/4396781/dewiss_datenbanken_delphi-studien/collections/25H44TFI). Table 1 summarizes the method used to compile the primary studies.

**Table 1:** Delphi expert network (DEWISS) data resource on Delphi primary studies

Search process	Description
Databases searched	• Scopus, MEDLINE, CINAHL, Epistemonikos in April 2021
Search strategy	• Keywords “Delphi*” in title or abstract from 2016 to April 2021
Selection criteria	• English or German language • Original papers with Delphi studies
Data extraction	• Title-abstract screening by six researchers and research fellows in the Delphi expert network without verification by a second reviewer

The * symbol allows you to include other terms that are used in connection with the word Delphi, e.g. Delphi study, Delphi-study, Delphi-Verfahren, Delphi-Studie

The authors are part of the Delphi network and collaborated in creating the collection of Delphi primary studies, which was compiled using central databases in the health and social sciences (Scopus, MEDLINE via PubMed, CINAHL, and Epistemonikos). Original works in German or English published between January 1, 2016, and April 21, 2021, were searched for in the four databases using the search term “title:(delphi*) OR abstract:(delphi*).” Following this, the titles and abstracts were screened by one researcher to include the English- and German-language Delphi studies in the health and social sciences. A total of six researchers were involved in the screening process and were in close contact with each other.

### Inclusion criteria

Guidance on reporting Delphi studies in palliative care has been available since 2017 via the Equator Network [15]. It is possible that this has an influence on the reporting and practice of Delphi studies in the health sciences and differences are visible from previous systematic reviews of Delphi study reporting [6, 7, 16]. For this reason, publications starting in 2018 are included in this review.

To identify classic or modified Delphi procedures in the health sciences aimed at establishing consensus, the DEWISS data resource of 7044 Delphi primary studies were filtered using the search term “(Title fields: health OR Abstract: health) AND (Title fields: consensus OR Abstract: consensus) AND Title fields: Delphi.” The inclusion criteria in the subsequent full-text screening entail the presence of a Delphi primary study on a health-related topic published in German or English. In regard to Delphi variants, both classic and modified Delphi studies were included when the aim was to find consensus and the consensus criteria were defined (Table 2). Studies that conducted a Delphi study in combination with another study, e.g., a previous systematic review to develop the Delphi questionnaire, were also included.

**Table 2:** Inclusion and exclusion criteria

	Inclusion criteria	Exclusion criteria
Article type	Original paper of a Delphi study	Systematic reviews, study protocols, and commentaries (on Delphi studies)
Language	German, English	A language other than English or German
Topic	Relevance to health	Studies on economic and technical topics not related to health
Delphi variants	Traditional or modified Delphi study that has the constitutive characteristics of a Delphi: 1) Survey of several people with specialized knowledge (known as experts) (e.g., operational knowledge, experiential knowledge, functional knowledge, contextual knowledge) 2) Carrying out at least two survey rounds or the option to respond at least two times 3) Feedback, the (interim) results are presented to the respondents with a possibility to respond	Study types other than Delphi studies, if these were not conducted in combination with a Delphi study
Study aim	Finding consensus, whereby criteria are defined according to which the consensus was determined	It is explicitly stated that reaching a consensus is not the aim of the Delphi study

**Fig. 3:**
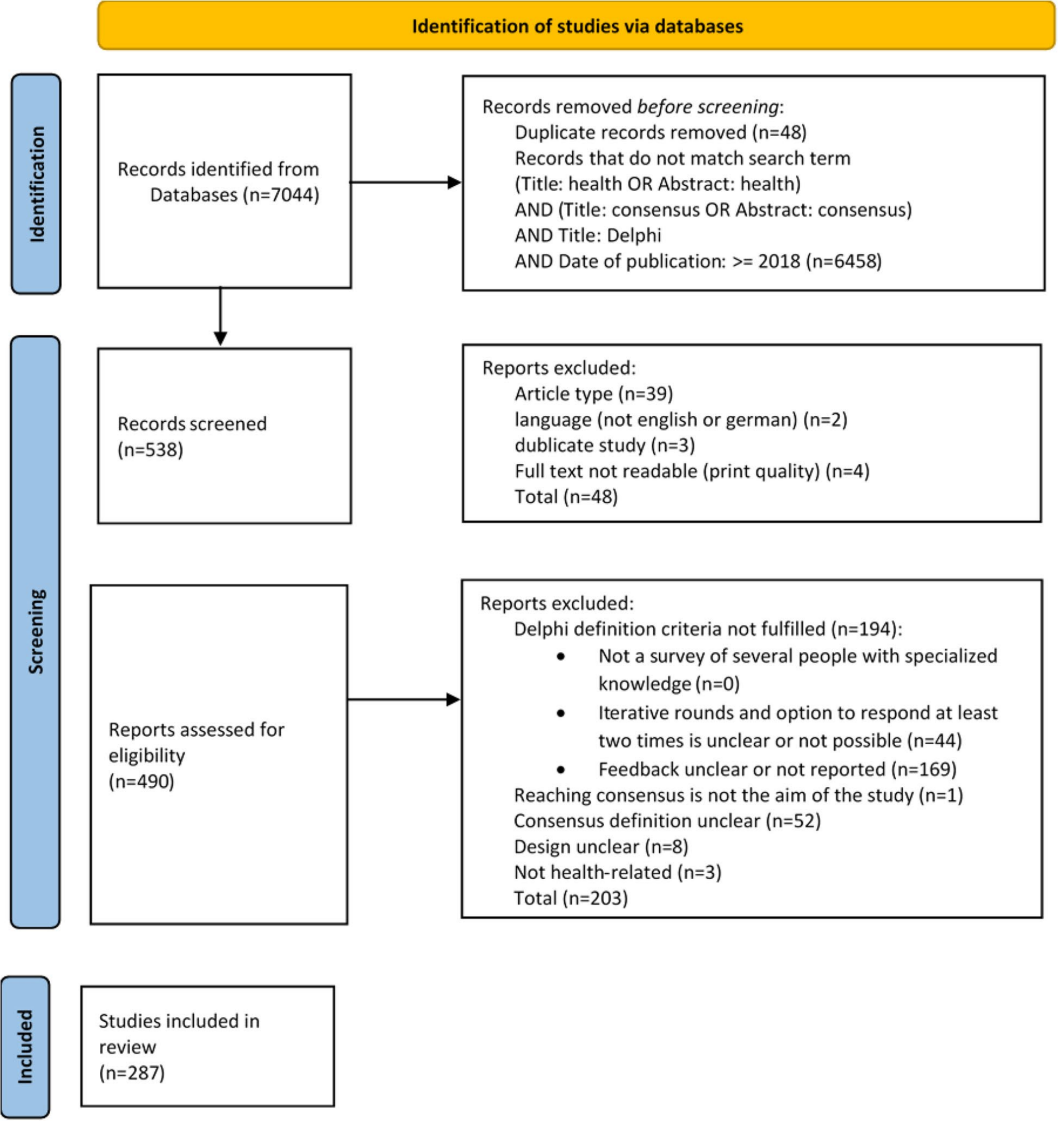
Literature screening process

Any publications that did not meet one or more of the three constitutive characteristics (expert survey, iterative rounds, feedback) or did not report on them were excluded. According to our definition, studies that survey experts in multiple rounds but do not share the results of previous rounds with the experts during the survey process are not Delphi studies. Here it must be noted that this decision is made solely on the basis of the publication leaving the possibility that some studies were excluded even though they have all the characteristics of a Delphi. The reason for exclusion was documented (Fig. 3). The full texts were screened by one of the authors (JS). The authors conferred with each other when uncertainty arose about the inclusion of individual studies. The authors then discussed and agreed whether the study should be included or excluded.

### Data extraction and analysis

A qualitative analysis strategy, namely qualitative content analysis, was selected to assess the publications [72]. This enables the analysis of large datasets and the inductive identification of categories based on the material and a picture of their range. Quantification of the results is also possible. Deductive main categories were formed as the basis for the qualitative analysis. These are the three factors that allegedly influence judgment formation: (1) the expert panel, (2) the questionnaire design, and (3) the process and feedback design. Using these deductive main categories, the research on the conduction of Delphi studies was documented, inductively filled in, and supplemented in terms of content. We have divided the inductive subcategories into two levels, with the second level further differentiating the first level. The formation of inductive subcategories was done by the first author using a random selection of approximately 10% of the included publications. This complies with current guidance for qualitative content analyses [72].

The category system was then discussed and revised with three of the seven members of the DEWISS network from the “Reporting Guideline” working group to assess the completeness and clarity of the definitions. The revised category system was verified anew using a random selection of 10% of the included publications. Some of the subcategories turned out to be difficult to analyze or did not yield much information due to a lack of uniformity in the reporting. For example, it was not possible to comparatively determine how much consensus was achieved in the Delphi studies because it was often unclear how many items had been analyzed in total. Also, the terms “question” and “item” were sometimes used synonymously or imprecisely.

Finally, the first author formulated explanations and added examples for all subcategories, and then discussed and finalized the category system again with the second author.

### Final category system

The final category system entailed 4 deductive main categories, 22 subcategories for the first level, and 58 subcategories for the second level (Table 3). The analysis of all of the publications was carried out by one researcher (first author) using Microsoft Excel.

**Table 3:** Category system to analyze the Delphi studies

No	Main category	Subcategory 1	Subcategory 2
1	General aspects	Area	1. Clinical patient care = diagnosis and therapy of diseases in inpatient settings, e.g., ID5^a^ 2. Healthcare services/public health = management of diseases, availability of care, access to healthcare, policy implication, e.g., ID35 3. Medical education = teaching and studying in health science programs, competencies of healthcare professionals, e.g., ID83 4. Methodological health research = methods in healthcare, research on research, e.g., ID134
2	General aspects	Delphi variant	1. Classic = reported as a classic Delphi study or not reported as modified Delphi study 2. Modified = reported as modified Delphi study
3	General aspects	Consensus criterion for rating scales	1. Standardized measure of dispersion = e.g., coefficient of variation, interquartile range, standard deviation 2. Standardized measure of central tendency = e.g., median, mean 3. Percentage agreement (one scale point) = proportion of agreement with a value, e.g., 70% vote for 5 on a 5-point scale 4. Percentage agreement (adjacent scale points) = proportion of agreement with two adjacent values, e.g., 70% vote for 3 or 4 on a scale of 1–5 5. Percentage agreement (other conditions) = other criteria for measuring percentage agreement, e.g., less than 15% vote 1 or 2 and at least 70% vote 6 or 7 on a 7-point scale or proportion of agreement within specific subgroups 6. Percentage agreement (unclear) = unclear definition of consensus, e.g., unclear which scale items were used to measure percent agreement 7. Dependency analyses = e.g., Kendall’s coefficient of concordance, Spearman's rho 8. Other criteria = e.g., number of outcomes predefined, content validity index, RAND/UCLA disagreement index, diversity of responses
4	General aspects	Percentage level consensus	Reported percentage level consensus in, e.g., 75%. Criteria may differ between Delphi rounds. In this case, all criteria were noted
3	Panel of experts	Sampling strategy	1. Snowball sampling = researcher relies on participant referrals to recruit new participants, e.g., recruiting colleagues from your own network 2. Purposive sampling = researcher seeks out participants with specific characteristics, e.g., recruiting researchers on the topic of artificial intelligence in clinical patient care 3. Purposive quota/random sampling = researcher randomly selects cases from within several different subgroups/quota, e.g., random selection of a number of the identified researchers on the topic of artificial intelligence in clinical patient care 4. Pool from a previous project or register = researchers select cases from a previous project or register, e.g., participants from a previous study 5. Convenience sampling = the authors reported to have selected according to convenience sampling, e.g., researcher gathers data from whatever cases happen to be convenient 6. Open calls = researchers recruit through open calls, e.g., through professional societies, regional networks, and advertisements on social media platforms
4	Panel of experts	Number of participants first round	Reported number of experts completing the first survey round
5	Panel of experts	Number of participants last round	Reported number of experts completing the last survey round
6	Panel of experts	Heterogeneity of expertise	1. Homogeneous = only one group of participants, e.g., nurses 2. Heterogeneous = the panel consists of participants from different disciplines and/or subject areas, e.g., nurses, care managers, nursing researchers 3. Heterogeneous including everyday life experts (e.g., patients) = the panel consists of participants from different disciplines and/or subject areas including affected persons, e.g., patients, patient representatives, affected persons
7	Panel of experts	Scope	1. national = one country, e.g., Germany 2. international = two or more countries without local scope, e.g., Germany and South Africa 3. Local = local cross-national focus, e.g., German-speaking region 4. Regional = specific region in one country, e.g., central Berlin
8	Questionnaire design	Survey software	Name of the digital platform for conducting the survey rounds, e.g. SurveyMonkey, LimeSurvey, Google forms, Microsoft Excel
9	Questionnaire design	Question types first Delphi round	1. Closed questions = questions with one or more answer options to choose from, e.g., rating, ranking, and multiple-choice questions 2. Closed questions with the possibility to comment = questions with one or more answer options to choose from and the possibility to comment on answers, e.g., including the option to reformulate or suggest new items 3. Open-ended questions = exclusively questions with free-text fields, e.g., in a first qualitative Delphi round
10	Questionnaire design	Number of items first Delphi round	Reported number of items or questions of the first survey round. Subdivision according to items and questions was not given in every case
11	Questionnaire design	Question types last Delphi round	1. Closed questions 2. Closed questions with the possibility to comment 3. Open-ended questions
12	Questionnaire design	Number of items last Delphi round	Number of items or questions of the last survey round. Subdivision according to items and questions was not given in every case
13	Questionnaire design	Width of rating scales	Width of rating scales, e.g., 4-point scale (1 = strongly agree, 4 = strongly disagree). If the response options or scale endpoints are not reported or are unclear, only the scale width is noted, e.g., 4-point scale
14	Questionnaire design	Rating scale, evasive category	Use of an evasive category, e.g., “unsure” or “don’t know”—option to answer, option to answer “Absent” due to a lack of perceived expertise. Recorded as reported or not reported
15	Questionnaire design	Randomization of questionnaire content	Randomization of question blocks, questions in question blocks, answer options in questions, e.g., through the survey software randomly assigning respondents. Recorded as reported or not reported
18	Process and feedback design	Timing of consensus definition	1. A priori = determined before the Delphi round 2. A posteriori = determined after the Delphi round
19	Process and feedback design	Method or literature reference for the analysis of qualitative data	1. Content analysis 2. Thematic analysis 3. Inductive approach 1–3 = reported method as mentioned in the text, e.g., thematic analysis 4. Other = e.g., grounded theory, quantitative analysis
20	Process and feedback design	Feedback designed to reconsider the judgments	1. Group response or summary = summary of qualitative date, e.g., comments from open-ended questions, or summary of quantitative data, e.g., statistical feedback of results from closed-ended questions 2. Group response or summary of different groups of participants = peer feedback of one or different groups of participants, e.g., caregivers received feedback from patients or only from caregivers 3. Individual response = display the respondent's answer from the previous round
21	Process and feedback design	Termination criterion	1. Consensus reached = achieving consensus in the Delphi study on all or the majority of the issues 2. Number of rounds = terminate the Delphi study after a predefined number of rounds, e.g., after two rounds of voting 3. Stability of judgments = terminate the Delphi study if the judgments are stable, e.g., determined through interquartile range, changes in mean scores 4. Other criteria = other criteria to terminate the Delphi study, e.g., when no new items are proposed, the judgments align, the response rate dropped below a certain value
22	Process and feedback design	Number of rounds	Reported number of survey rounds/iterations, e.g., three Delphi rounds

^a^The ID refers to the analyzed publication. An overview of the analyzed studies and results is shown in Additional file 1

## Results

The search for consensus Delphi studies in the health sciences in the database of Delphi primary studies yielded 538 hits. Forty-eight studies were excluded in the first step of full-text screening because they were not original papers on Delphi primary studies or did not meet other inclusion criteria (e.g., German or English language) (Fig. 3). For 40% (*n* = 194/490) of the studies it was unclear if a Delphi study with the constitutive characteristics (Table 2) had been carried out or the criteria for determining consensus remained unclear (Fig. 3). A total of 287 studies satisfying the inclusion criteria were included in the analysis (Additional file 1).

### General aspects

#### Area

Consensus Delphi studies in healthcare were assigned to four topical areas: clinical patient care (e.g., ID1, ID21, ID204, ID213),^1^ healthcare/public health (e.g., ID6, ID18, ID93, ID168), medical education (e.g., ID83, ID128, ID162) and methodological health research (e.g., ID134, ID164) (Table 4). The aims for reaching consensus include the development of guidelines and recommendations for the diagnosis and therapy of disease (e.g., ID11, ID170), the forecasting of healthcare needs and research priorities (e.g., ID67, ID199), the development of measuring instruments and validation of findings (e.g., ID180, ID191), agreement on definitions and terminologies (e.g., ID1, ID146) and defining competency profiles and curricula in healthcare (e.g., ID176, ID267).

**Table 4:** Main category “General aspects” of the Delphi studies included in the scoping review

No	Subcategory 1	Subcategory 2: Frequency % (*n*/287) and statistics
1	Area	1. Clinical patient care: 38% (*n* = 110) 2. Healthcare services/public health: 39% (*n* = 112) 3. Medical education: 14% (*n* = 40) 4. Methodological health research: 9% (*n* = 25)
2	Delphi variant	1. Classic: 57% (*n* = 165) 2. Modified: 43% (*n* = 122)
3	Consensus criterion for rating scales	1. standardized measure of dispersion: 20% (*n* = 57) 2. standardized measure of central tendency: 21% (*n* = 60) 3. Percentage agreement (one scale point): 14% (*n* = 40) 4. Percentage agreement (adjacent scale points): 45% (*n* = 129) 5. Percentage agreement (other conditions): 13% (*n* = 37) 6. Percentage agreement (unclear): 12% (*n* = 35) 7. Dependency analyses: 2% (*n* = 6) 8. Other criteria: 3% (*n* = 10)
4	Percentage level consensus	Mean (standard deviation): 73,1% (8,3) Minimum/maximum: 40%/100% Median: 75%

### Delphi variant

The classic Delphi technique is most often selected in the studies analyzed, but in 43% (*n* = 122/287) of the Delphi studies it is reported that a modified Delphi was carried out (Table 4). Among others, the modifications identified were an initial survey round with closed instead of open questions (e.g., ID5, ID71), face-to-face meetings of the participants, or a combination of anonymous survey rounds with group discussions (e.g., ID6, ID32, ID95), the integration of collecting and analyzing qualitative data (e.g., ID19, ID36), no meeting of the participants during the process (e.g., ID14), conducting the study as an online survey/e-Delphi (e.g., ID28, ID76) or defining the number of rounds a priori (e.g., ID16, ID42). What is viewed as a modification in these studies varies and is, in part, even contradictory. Sometimes it is not reported at all (e.g., ID8, ID16, ID89, ID206, ID279). For 80% (*n* = 231/287) of the Delphi studies, it is reported that the Delphi took place online. Meetings between the participants took place in 16% (*n* = 45/287) of the Delphi studies as part of the process. Specific Delphi variants were identified in individual cases, for instance, the argumentative Delphi (e.g., ID214), policy Delphi (e.g., ID31, ID73, ID150, ID214), real-time Delphi (e.g., ID111, ID241), or fuzzy Delphi (e.g., ID284).

#### Definition of consensus

Reporting the definition of consensus was stipulated as an inclusion criterion for the scoping review. Consensus was defined in 30% of the Delphi studies (*n* = 87/287) using statistical measures. Most frequently, in 81% (*n* = 232/287) of the Delphi studies, the consensus was defined as percentage agreement, which is understood differently among the Delphi studies, e.g., whether one or several adjacent scale points are used (Table 4). The cut-off values are generally at 70% (*n* = 87), 75% (*n* = 49) or 80% (*n* = 67). Some Delphi studies divide the strength of consensus according to different levels (e.g., ID73, ID118, ID142, ID150, ID155, ID177), whereby these assignments entail three to five levels (ID73: perfect consensus, consensus, wide agreement, majority, and large minority; ID118: high consensus, low consensus, no consensus; ID142: very high, high, moderate, low; ID150, ID155 and ID177: high, moderate, low, no consensus).

### Panel of experts

#### Sampling strategy

Ninety-four percent (*n* = 271/287) of the Delphi studies report how the participants were recruited, e.g., through purposive or snowball sampling (Table 5). In 62% (*n* = 178/287) of the Delphi studies the authors describe a concrete sampling strategy and 32% (*n* = 93/287) apply a combination of different strategies.

**Table 5:** Main category “Panel of experts “ of the Delphi studies included in the scoping review

No	Subcategory 1	Subcategory 2: Frequency % (*n*/287) and statistics
3	Sampling strategy	1. Snowball sampling: 29% (*n* = 82) 2. Purposive sampling: 78% (*n* = 225) 3. Purposive quota/random sampling: 2% (*n* = 6) 4. Pool from a previous project or register: 6% (*n* = 17) 5. Convenience sampling: 3% (*n* = 10) 6. open calls: 12% (*n* = 34) *Unclear or not reported: 6% (n* = *16)*
4	Number of participants first round	Mean (standard deviation): 60,2 (105,8) Minimum/maximum: 4/1014 Median: 31 *Unclear or not reported: 4% *(*n* = 12)
5	Number of participants last round	Mean (standard deviation): 46,8 (72,7) Minimum/maximum: 3/713 Median: 26 *Unclear or not reported: 5% (n* = *15)*
6	Heterogeneity of expertise	1. Homogeneous: 6% (*n* = 18)^a^ 2. Heterogeneous: 65% (*n* = 187) 3. Heterogeneous including everyday life experts (e.g., patients): 25% (*n* = 73) *Unclear or not reported:* 3% *(n* = *9)*
7	Scope	1. National: 51% (*n* = 145) 2. International: 36% (*n* = 102) 3. Local: 6% (*n* = 17) 4. Regional: 5% (*n* = 15) *Unclear or not reported: 3% (n* = *8)*

^a^Of these, three studies were only with patients

#### Number of participants (first and last round)

Around 60 experts on average participated in the first Delphi round and 47 in the final Delphi round. The studies vary widely from 4 (ID39) to 1014 (ID51) participants in the first Delphi round and from 3 (ID234) to 713 (ID51) in the last round (Table 5). In 83% (*n* = 239/287) of the Delphi studies the number of experts in the final Delphi round is the same size or smaller than in the first round. Often only the experts who completed the previous round are invited to participate in the subsequent rounds (e.g., ID1, ID24, ID49, ID73, ID147, ID207). However, there are also Delphi studies in which the panel is enlarged (e.g., ID16, ID70, ID71, ID95, ID111) or all of the experts are invited to participate, regardless of their participation in the separate Delphi rounds (e.g., ID2, ID27, ID28, ID62, ID124, ID205, ID233). Regarding the Delphi studies with the same or fewer number of participants in the final Delphi round as compared to the first, the expert panel in the final round is on average 19% smaller than in the first Delphi round.

#### Heterogeneity of the panel and scope

Recruitment was generally done according to criteria defining the expertise or the professional background of the experts (e.g., ID3, ID20, ID79, ID268). A homogeneous expert panel is reported by 6% (*n* = 18/287) of the Delphi studies, meaning that it consisted of only one expert group (e.g., ID7, ID33). In the analyzed publications, the heterogeneity of the panel is reported as a quality criterion for the studies (e.g., ID81) but the shape this takes differs. These panels are, as a result, multidisciplinary in their composition, meaning that different disciplines are present in one area of expertise (e.g., ID5, ID38, ID130), or they are transdisciplinary, meaning different areas of expertise represented, such as theory and practice (e.g., ID92, ID100, ID181). Affected populations, e.g., patients, are included in 27% (*n* = 76/278) of the Delphi studies. The scope of recruitment for the Delphi studies is 51% (*n* = 145/287) at the national level and 36% (*n* = 102/287) internationally (Table 5). For 3% (*n* = 8/287) of the publications, the specific regional focus remained unclear.

### Questionnaire design

#### Survey software

Approximately 30 different software programs were named as the online survey platform, whereby these are primarily designed for conventional population surveys and not explicitly for carrying out Delphi studies, e.g., Qualtrics (https://www.qualtrics.com), SurveyMonkey (https://www.surveymonkey.com), and Google Forms (https://www.google.com/forms/about/). Qualtrics software was used most often making up 19% of the cases (Table 6). In 39% (*n* = 111/287) of the Delphi studies the survey software was not identified or none was used if, for instance, the questionnaires were sent directly to the participants via email.

**Table 6:** Main category “Questionnaire design” of the Delphi studies included in the scoping review

No	Subcategory 1	Subcategory 2: Frequency % (*n*/287) and statistics
8	Survey software	1. Qualtrics: 19% (*n* = 55) 2. SurveyMonkey: 12% (*n* = 34) 3. REDCap: 7% (*n* = 21) 4. Google Forms: 5% (*n* = 15) 5. LimeSurvey: 4% (*n* = 12) 6. Other: 15% (*n* = 42) *Unclear or not reported: 39% (n* = *111)*
9	Question types first Delphi round	1. Closed questions: 10% (*n* = 28) 2. Closed questions with a possibility to comment: 66% (*n* = 189) 3. Open-ended questions: 22% (*n* = 63) *Unclear or not reported: 2% (n* = *7)*
10	Number of items first Delphi round*	*Closed questions (with a possibility to comment)* Mean (standard deviation): 60,6 (61,1) Minimum/maximum: 1/525 Median: 45 *Open-ended questions* Mean (standard deviation): 5,2 (6,1) Minimum/maximum: 1/26 Median: 3 *Unclear or not reported: 17% (n* = *50)*
11	Question types last Delphi round	1. Closed questions: 44% (*n* = 125) 2. Closed questions with a possibility to comment: 50% (*n* = 144) 3. Open-ended questions: 0% (*n* = 0) *Unclear or not reported: 6% (n* = *18)*
12	Number of items last Delphi round*	Mean (standard deviation): 39,1 (43,5) Minimum/maximum: 1/289 Median: 24 *Unclear or not reported: 23% (n* = *66)*
13	Width of rating scale	2-point scale: 11% (*n* = 31) 3-point scale: 9% (*n* = 27) 4-point scale: 10% (*n* = 28) 5-point scale: 40% (*n* = 116) 6-point scale: 2% (*n* = 5) 7-point scale: 11% (*n* = 33) 9-point scale: 20% (*n* = 56) 10-point scale: 4% (*n* = 12) 11-point scale: 1% (*n* = 4) *Unclear or not reported/not applicable: 5% (n* = *14)*
14	Rating scale, evasive category	Reported: 20% (*n* = 56) *Unclear or not reported: 80% (n* = *231)*
15	Randomization of questionnaire content	Reported: 2% (*n* = 7) *Not reported or not applicable:* 98% (*n* = 280)

*In one Delphi study the mean value was based on the given range of items because the study participants had received differing numbers of items

#### Question types and number of items (first and last Delphi round)

Standardized and open questions were used in the questionnaires, with the number of standardized items increasing with the number of rounds. A total of 10% (*n* = 28/287) of the Delphi studies reported asking only closed questions or did not report on the options to comment openly in the first round; the same applied to 44% (*n* = 125/287) of the Delphi studies in regard to the final round (Table 6). The number of standardized questions ranged from only a few (e.g., ID132, ID136) to several hundred (e.g., ID55, ID56, ID101) in the first Delphi round, with the observation that there is a tendency to decrease with each Delphi round.

#### Rating scale (width and evasive category)

In 79% of the Delphi studies (*n* = 226/287) it was reported that an odd-numbered rating scale was used to capture the individual judgments. Five-point Likert scales were most commonly used in 40% (*n* = 116/287) of the Delphi studies (Table 6). Sometimes over the course of a Delphi study, other rating scales with different scale widths are used (e.g., ID13, ID73, ID114, ID124, ID143, ID251). No Delphi study reported more than 11 scale points. Differences can be seen in the identification of the scale gradations. The scales or response options are either offered verbally (e.g., “strongly agree,” “strongly disagree” (ID67)) or verbally and numerically (e.g., 1 = disagree, 5 = agree (ID11)). Overall, in 20% (*n* = 56/278) of the Delphi studies it is reported that an evasive category could be selected. These are presented either as a separate response option (e.g., ID1, ID20, ID45, ID51, ID260) or a middle category (e.g., ID47, ID56, ID66, ID81, ID152).

#### Randomization of questionnaire content

The content of the Delphi study questionnaires was organized on the level of the questionnaire itself, e.g., in the sequencing of the content according to topic (e.g., ID24, ID34, ID52, ID56), and on the level of the questions, e.g., when response options were sorted according to frequency in the previous round (e.g., ID14, ID237, ID236). Only 2% (*n* = 7/287) of the Delphi studies stated that the content of the questionnaire—the topic segments or response options—was randomized.

### Process and feedback design

#### Timing of consensus definition

The point in time at which consensus was defined is not reported in 61% (*n* = 176/287) of the Delphi studies. In 36% (*n* = 103/287) of the Delphi studies the authors state that consensus was defined in advance (Table 7).

**Table 7:** Main category “Process and feedback design” of the Delphi studies included in the scoping review

No	Subcategory 1	Subcategory2: frequency % (*n*/287) and statistics
17	Timing of consensus definition	1. a priori: 36% (*n* = 103) 2. a posteriori: 3% (*n* = 8) *Unclear or not reported: 61% (n* = *176)*
18	Method and/or literature reference for the analysis of qualitative data	1. Content analysis: 14% (*n* = 41) 2. Thematic analysis: 13% (*n* = 38) 3. Inductive approach: 1% (*n* = 3) 4. Other: 1% (*n* = 3) *Unclear or not reported: 62% (n* = *178)* *Not applicable (n* = *26): 9%*
19	Feedback design to reconsider the judgments	1. Group response or summary: 93% (*n* = 268) 2. Group response or summary of different groups of participants: 6% (*n* = 18) 3. Individual response: 46% (*n* = 132) *Unclear or not reported: 0% (n* = *0)*
20	Termination criterion	1. Consensus reached: 23% (*n* = 66) 2. Number of rounds: 30% (*n* = 85) 3. Stability of judgments: 9% (*n* = 26) 4. Other criteria: 1% (*n* = 4) *Unclear or not reported: 46% (n* = *132)*
21	Number of rounds	Mean (standard deviation): 2,8 (0,8) Minimum/maximum: 2/8 Median: 3 *Unclear or not reported:* < *1% (n* = *1)*

#### Data analysis and feedback design to reconsider the judgments

In almost all of the Delphi studies (93%(*n* = 268/287)) it was reported that feedback was given to the participants in the form of information on the statistical group responses and/or a summary of the qualitative or quantitative data. It is also the case, where over half of the Delphi studies included free-text fields in the questionnaire (Table 6), and 62% (*n* = 178/287) of the Delphi studies do not report the analytical method applied to the qualitative data (e.g., ID2, ID20, ID71, ID123). For 46% (*n* = 132/287) of the Delphi studies, it was also indicated that the participants were able to see their responses from the previous round (e.g., ID106, ID110, ID243). Peer feedback from one or more participant groups was reported by 6% of the Delphi studies (e.g., ID58, ID247, ID255).

#### Termination criterion and number of rounds

In 30% (*n* = 85/287) of the Delphi studies the survey process was terminated after a previously defined number of rounds. Three rounds were held in 54% (*n* = 156/287) of the Delphi studies, and two rounds in 36% (*n* = 104/287). The highest number of rounds was eight (ID71). The criterion for terminating the Delphi study remained unclear for around half of the Delphi studies (e.g., ID4, ID89, ID151).

## Discussion

The scoping review analyzes the conduction of consensus Delphi studies in the health sciences based on publications. As documented in previous reviews [14–16], there were many diverse modifications such that it is impossible to refer to *the* Delphi technique. Because these modifications are seldom reflected on or justified, negative effects on the quality and ultimately on the acceptance and implementation of the results cannot be ruled out. This can be seen in the factors of expert panel, questionnaire design, and process and feedback design.

### Expert panel

The findings suggest that it can be difficult to acquire experts for Delphi studies and to retain them over multiple rounds. Indications of this are the sampling strategies and decreasing numbers that have already been discussed in other reviews [7, 14]. Reasons for dropping out remain unclear and were usually not reflected in the Delphi studies analyzed here. However, this is an important but under-researched issue for the validity of the results. Gargon et al. [73] suspect that the “attrition of participants could mean that people with minority opinions drop out of the Delphi study, leading to an overestimation of consensus.” High dropout rates can therefore have a negative impact on the credibility of the process and the validity of the consensus [11]. It also remained unclear for which reasons a Delphi study was terminated [14]. Ideally, termination occurs when consensus or agreement regarding the dissent has been achieved and/or the judgments are stable, but pragmatic reasons are also conceivable when, for instance, too many experts drop out or the resources for further rounds are lacking.

The goal of Delphi studies is to include different expert groups [25, 32]. Yet the numerical ratio between expert groups often remains unclear. Complicating this is that expert groups are defined and differentiated in varying ways. The wording is not always consistent either. For example, the participants in a Delphi are usually referred to as experts, but sometimes also as panelists [2]. In general, one of the greatest challenges in Delphi studies is the identification and recruitment of relevant experts [14, 56].

As a result, it is impossible to make any reliable claims about the influence of a panel's composition on the results. Especially when lifeworld experts are included, such as patients or their family members, it may be necessary, due to the educational backgrounds, to adapt the technical terminology or provide additional information on the current state of research so that these participants are not overwhelmed and to enable a well-considered judgment [39]. Otherwise, there is the risk that satisficing effects or drop-out rates [28, 29] will be more probable for this group. To reduce the probability that important perspectives are lost, Boel et al. [74] recommend, based on a methodological experiment, always inviting all of the experts to each Delphi round regardless of their participation or nonparticipation in the previous round. Their recommendation is justified by their study in which the response rate for the "all-rounds group" was higher (61%) compared to the “respondents-only group” (46%) [74]. Another strategy to reduce attrition is to send reminders [56]. It is also possible to contact experts personally and send personalized emails, which can have an additional positive effect on the response rate [73].

### Questionnaire design

In contrast to a review from the field of histopathology [16], the results here show a clear preference for five-point rating scales to record consensus. Regardless of the exact number of scale points, the use of an odd-numbered rating scale means that respondents have an evasive option in the ‘middle.” The effect of the number of scale points on judgments in Delphi studies has not been conclusively clarified [14]. With the mental model in mind (see “Theoretical insights on judgment formation in consensus Delphi studies” section), it could be argued that the experts can avoid taking a clear position and engaging deeply with the questions by using middle categories. This could be compounded if, in the case of an online Delphi, questions are programmed such that experts cannot skip over them but rather must submit an answer. This could mean that in the worst-case scenario, the results of Delphi studies with a high degree of satisficing behavior by the participants reflect a consensus of “collective ignorance” or collective uncertainty [14], rather than the wisdom of the crowd [22].

To maintain the motivation of the experts despite this, Delphi practitioners appear to want to keep the time and cognitive effort as low as possible, thus also making satisficing effects less likely [28]. An indication of this is the tendency to shorten the questionnaire as the Delphi procedure progresses. However, it is impossible to verify the stability of the judgments when items for which there is consensus are taken out [5].

### Process and feedback design

As evidenced in earlier reviews [2, 7, 16], the design of the feedback is reported unclearly or not at all. In particular, the method used for qualitative analysis remains vague, as does whether or not all of the expert groups have participated equally in the arguments. After the writing of this review, the AQUA (Argument-based QUalitative Analysis strategy) was published, offering for the first time a strategy to analyze open-ended responses in Delphi studies [65]. It remains to be seen if proposals of this nature have an influence on how free-text responses are handled in Delphi studies.

### Research practice and the quality of the results of Delphi studies

The analysis shows a wide variance in how consensus Delphi studies are conducted. This diversity has been a consistent topic of discussion for decades in critical assessments of Delphi studies [15, 75, 76]. At the same time, the possibilities offered by modern software [14] and new scientific standards, such as the inclusion of affected parties [77] or the combination of methods or triangulation [78], evoke the necessity for further methodological development of the Delphi technique. However, there is the risk that such developments are applied uncritically or without reflection because they are currently “state of the art.” As a result, the combination of quantitative and qualitative data in Delphi studies has been done in a manner that is hardly systematic, whereby potentially significant information and insights are lost [65]. Furthermore, the participation of affected persons also goes without careful consideration in terms of methodology.

Overall, the impression is reinforced that the conduction of Delphi studies as a form of research is pragmatic in approach. This is suggested by the unthought-out number of Delphi rounds, the incomprehensible removal of items, and the lack of analyses regarding reasons for the absence or stability of judgments. Beiderbeck et al. [56] recommend considering a priori criteria for stopping the Delphi process (e.g., when judgments are stable). However, as shown in this review, the traditional measure of stability is rarely used. Another option could be to measure expert contribution [79], e.g., by the number of comments if this falls below a certain level. The example shows that it is essential for the applicability of quality criteria that they are feasible for the researchers and comprehensible for the reader of the Delphi study publications [79].

The Delphi technique's enduring popularity, which is even increasing in healthcare [27], could persist precisely because this method can be “pragmatically” adapted. That said, though, pragmatically conducted research cannot take place at the cost of systematic method, transparency, and quality [1, 2]. Otherwise, there is the danger that the consensus has no validity in practice [14].

A first step to make Delphi studies potentially more comparable with each other and to support their application would be interdisciplinary and cross-disciplinary agreement on a recommended definition of consensus. Valid definitions from medicine could serve as proposals allowing for consensus to be subdivided into three categories, e.g., high (70% agreement) moderate (60% agreement), and low consensus (50% agreement) [17] or strong consensus (95% agreement), consensus (75% agreement), and majority agreement (50% agreement) [80].

### Advice for Delphi practitioners to ensure the quality Delphi studies

Based on the analysis of research practice and given the current state of research on Delphi studies, there are, in our opinion, various suggestions for Delphi practitioners in the health sciences regarding the three factors analyzed here to ensure and improve the quality of study results. We view these suggestions as a contribution to the discourse on the development of practical guidelines for transparent and critical conduction of Delphi studies.

1. Expert panel

• Define the relevant expert groups and see that there is a balanced ratio in order to avoid potential distortions due to uneven ratios.

• Critically consider the heterogeneity of the expert panel, particularly in regard to its relevance for the questionnaire and feedback design.

• Be sure that the number of participants in each expert group is a two-digit number. Depending on the research field, it may be necessary at first to invite five to ten times the number of experts needed for each expert group to retain sufficient numbers over multiple rounds. Consider how participants' motivation can be maintained across all expert groups.

2) Questionnaire design

• Develop the initial questionnaire based on the current state of research and, if applicable, other preceding empirical studies. Include not only representatives from the relevant expert groups during development to test the content and its comprehensibility but also methodological experts to ensure the Delphi study’s practicability and the quality of its results.

• When designing the questionnaire, bear in mind that answering some of the questions is cognitively demanding so attention should be paid to the overall length and complexity of the questionnaire. Consider randomizing the questions if the questionnaires are lengthy and there is a sufficient number of participants.

• Reflect on the personal backgrounds and competencies of the experts. If dissimilarities exist, support the experts so that all of them can participate fully. For example, provide clear information on the state of research.

• Try to motivate the experts to produce explicit judgments (e.g., for or against a specific action). Consider if a middle category on a rating scale really makes sense. An alternative would be to weigh the option of a separate evasive category (e.g., “don't know”).

• We recommend only recording qualitative and quantitative data in a Delphi study if these are systematically analyzed and the findings are combined or triangulated.

3) Process and feedback design

• Avoid modifications to the Delphi technique that have not been critically considered or put to the test previously. However, if modifications are necessary, make them in a transparent manner and justify them.

• Define the criterion for consensus. A cut-off value of less than 70% is unusual in the health sciences.

• Consider when to end the Delphi study. If you set the number of Delphi rounds or a target corridor in advance, share this with the experts so that they can better estimate the time needed. In regard to participant motivation and securing results, we recommend holding a maximum of three Delphi rounds.

• Follow published guidance on reporting Delphi studies, e.g., DELPHISTAR (Delphi studies in social and health sciences—recommendations for an interdisciplinary standardized reporting) [81].

• Evaluate the Delphi study or integrate evaluative elements into the study, e.g., questions about which feedback information was considered or about the time needed to participate in the Delphi study.

## Limitations

The scoping review examines research practices involving Delphi studies in the health sciences. Since this paper focuses on consensus-oriented Delphi studies, the results here may only be limitedly transferable to Delphi studies pursuing other aims. The publications of the Delphi studies were screened and analyzed by one person, whereby the ongoing discussions within the research team and with scientists from the Delphi expert network (DEWISS) should have reduced this bias. There could be biases because only the publications were analyzed and not the Delphi studies themselves and, as a consequence, the findings here depend on the quality of the reporting. A selection bias may also exist because only available German and English full texts were analyzed.

## Conclusion

Research practice shows that consensus Delphi studies in healthcare are carried out in different ways, whereby the approach can sometimes be described as pragmatically based research. Due to unclear reporting and variance in study design, Delphi studies are currently only comparable and evaluable to a limited extent. Nevertheless, it is clear that Delphi studies play an important role in a variety of health science issues, such as curriculum or guideline development, definition and terminology, and prioritization of research needs, possibly because of their pragmatic approach. In our view, the challenge of uniform criteria for conducting and reporting lies in increasing the comparability of Delphi studies with each other and ensuring the quality of study results while at the same time allowing for the flexibility of the Delphi technique and innovations.

## Supplementary Information

Additional file 1: Overview of the analyzed publications and results of the qualitative content analysis.

## Data Availability

The dataset supporting the conclusions of this article is included within the article and its additional file.

## Footnotes

1 ID refers to the analyzed publication. An overview of the studies analyzed and the results is presented in Additional file 1.
